# Exact linearization and control of a mobile robot for the inspection of soil resources in *Solanum tuberosum* crops

**DOI:** 10.3389/frobt.2024.1459902

**Published:** 2024-09-13

**Authors:** Álvaro Pulido-Aponte, Claudia L. Garzón-Castro

**Affiliations:** Engineering Faculty, Research Group CAPSAB, Universidad de La Sabana, Campus del Puente del Común, Chía, Cundinamarca, Colombia

**Keywords:** differential robot, exact linearization, control, non-holonomic restrictions, agricultural applications, *Solanum tuberosum*

## Abstract

In recent years, the development of robots for agro-industrial applications, such as the cultivation of *Solanum tuberosum* potatoes, has aroused the interest of the academic and scientific communities. This is due, at least in part, to the complexity of modeling and robustly controlling some dynamics inherent to nonlinear behaviors normally attributed to the different technologies associated with the movement of these autonomous vehicles and their non-holonomic constraints. The different nonlinear dynamics of mobile robots are usually represented by state-space models. However, given some equilibrium and stability characteristics, the implementation of effective controllers for the robust parametric tracking and variation problem requires techniques that allow the operability of robots around regions of stable equilibrium. Feedback linearization control is one such technique that attempts to mathematically eliminate nonlinear expressions from the plant model. However, this technique requires an observable and controllable mathematical model. If there is some relationship between the model inputs and a controlled output that allows the relative degree of the control law to be determined, the controller design and implementation are posed as a linear issue. Flat filters developed from the generalized proportional integral control approach are an alternative that could facilitate the design of controllers for these linearized systems. From these flat filters, it is possible to obtain the transfer function of a controller without relying on the derivatives of the system output. This work proposes the design of a controller via exact linearization and its equivalent flat filter for a robot inspector of the soil resource of *S. tuberosum* crops in the department of Cundinamarca, Colombia. The actuator motion constraints resulted in a robot with two degrees of mobility and one non-holonomic constraint. Numerical validation of this system suggests that it can be an effective solution to the problem of tracking control at changing references by providing a system capable of navigating through crop rows. The results suggest correct tracking for linear and circular trajectories. However, the control lacks the ability to track spiral-type trajectories.

## 1 Introduction

Autonomous Mobile Robotics (AMR) has been a topic of interest in recent years due to the boom in different technological tools that have facilitated the development of robots for multiple domestic and industrial applications (Andronie et al., 2022). Specifically, in the framework of precision agriculture, specialized instrumentation and communication networks have enabled the development of specialized robots for some agricultural tasks (Abu et al., 2022). The above proposes a potential favorable impact on the resolution of various issues caused by the over-demand for some food products. In their production, this causes inefficient management of natural resources, excessive use of chemicals that affect the environment and human health, and high economic costs of food production, among others (FAO, 2021; Luthra et al., 2022; Salamanca Castillo, 2020; Ayala Garces, 2022; Acevedo-Osorio et al., 2017; Pérez-Ortega, Bolaños-Alomia, and da Silva, 2022). The *S. tuberosum* potato (*S. Tuberosum)*, for example, is one of the most consumed foods in the world, and its growing demand is latent, which represents a challenge for environmental, economic, and sanitary sustainability in the medium term (FEDEPAPA, 2020; Van der Hammen et al., 2002). In the last decades, some works have suggested that a timely inspection of the available soil resource could help in the phenological prediction of crops, especially those that fruit below ground, as is the case of *S. tuberosum* (Antonio, 2022; Flores-Magdaleno et al., 2014). Although the development of robots has partially facilitated some processes, such as planting, harvesting, irrigation, and weeding in *S. tuberosum* crops, little has been explored in relation to the implications that could lead to a robot inspector of the available soil resource and its potential impact on the mitigation of the problems (Tein et al., 2014; Oviedo-Chávez et al., 2023).

The implementation of paradigms associated with AMR has generated different engineering challenges for each of the possible stages of agricultural robot development (Dutta et al., 2021). In this sense, robust control, for example, is one of these stages and relies heavily on simple but accurate mathematical models to represent complex dynamics, generally of a nonlinear nature (Abbaspour et al., 2019). Although in the case of AMR there are actuator configurations that facilitate robot locomotion, as in the case of differential robots, they also have limitations due to non-integrable speed restrictions, also known as non-holonomic restrictions (Tchoń and Ratajczak, 2021). These constraints of Euler-Lagrange mechanics have a direct effect on the generalized coordinates of the robot’s position and, therefore, on its maneuverability (Náprstek and Fischer, 2021).

On the other hand, the general requirements given by the multiple applications of robots have led to the proposal of different classical control approaches to address issues such as regulation and tracking desired trajectories (Zangina et al., 2020). These approaches include open-loop strategies with preset motions and feedback control strategies that integrate techniques such as odometry with proportional-integral-derivative (PID) controllers, fuzzy logic, and feedback linearization control (Zangina et al., 2020; Saxena et al., 2024). The latter, highly dependent on the robot model, eliminates its nonlinear expressions and, from an input-output relationship given by a relative degree, imposes control dynamics (Spong et al., 2020). Although the feedback linearization technique offers some implementation advantages when addressing nonlinear control problems with linear control techniques (Martins et al., 2021; Alyoussef and Kaya, 2019). This technique also presents some limitations in terms of precise mathematical knowledge of the output, the reference, and its derivatives, depending strictly on the relative degree of the controller (Alyoussef and Kaya, 2019). Flat filters designed from the generalized proportional integral (GPI) control approach can complement the exact linearization control strategy by obtaining transfer functions that do not generate asymptotic observer dependence. The relative ease suggested by the implementation of these linear controllers is represented by algebraic polynomials and transfer functions. This leads to an exploration of the exact linearization and its potential applications in robots for the inspection of the soil resource in *S. tuberosum* crops. One of the tasks of these robots includes tracking ground trajectories for sampling soil nutritional variables. Therefore, the objective of this work is to propose the design of a controller based on the exact linearization of the direct kinematics model of a robot for the inspection of the soil resource in *S. tuberosum* crops in the department of Cundinamarca, Colombia. The robot consists of four geared motors in differential configuration. The model represents the motion of the robot as a function of its velocities and generalized position coordinates. The motion constraints of the actuators result in a robot with two degrees of mobility and one non-holonomic constraint. This paper is organized as follows: Section 2 provides an overview of the robot and contextualizes the tasks in terms of trajectory tracking, Section 3 discusses preliminary concepts related to the mathematical model of the robot and its motion constraints. In Section 4, a feedback linearization control of the robot is presented. In Section 5, the discussion is presented and finally concluded in Section 6.

## 2 Robot general description

The first version of the robot was developed by the Process Control and Automation Research Group of the Universidad de La Sabana (CAPSAB). The robot consists of a platform that integrates hardware and software. Details of the software were reported in (Pulido-Aponte and Garzón-Castro, 2024). As for the hardware, briefly, the robot is composed of 4 (four) gear motors (one for each wheel) with a maximum speed equal to $30\;min^{-1}$ and torque of 2.5Nm in differential configuration. The wheels have a diameter of $8^{\prime \prime }$ and, therefore, the maximum speed of the mobile is $19.15\frac{m}{\min}$ . The overall dimensions of the platform are 0.3 × 0.2 × 0.4 (m).

The main task of the robot is to navigate through the rows of an *S. tuberosum* crop while recording the nutritional variables available to the plants. The platform satisfies both hardware and software requirements given the climatic and topographic conditions of the region of Cundinamarca, Colombia.

## 3 Preliminaries

This section presents some concepts and definitions that lead to a mathematical model that represents the kinematics of the robot described in the previous section. For the purposes of the model, a differential mobile robot with concentric axes and perfectly circular, undeformable wheels perpendicular to its plane of motion was assumed. For this example, the model excludes endogenous and exogenous disturbances that may occur during vehicle operation.

### 3.1 Kinematic model considerations

**FIGURE 1:**
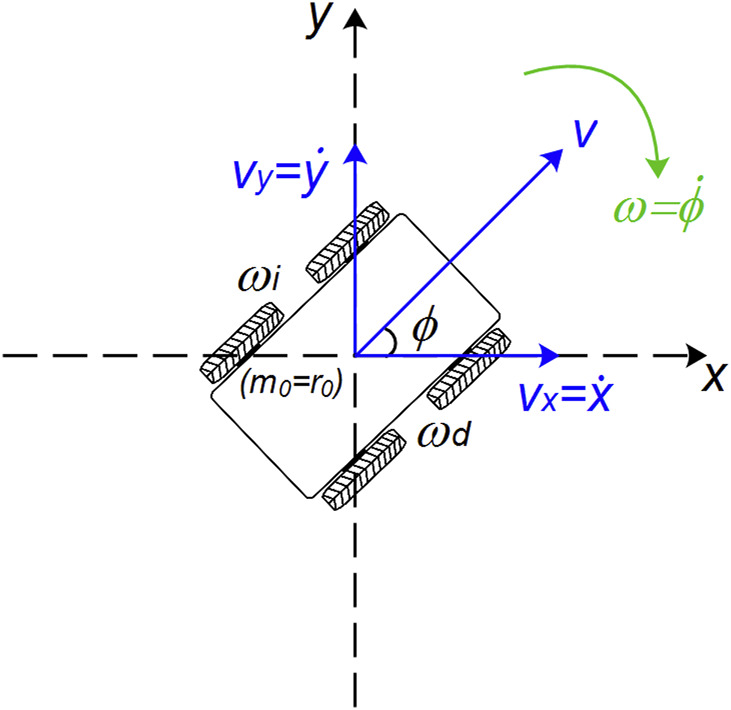
Direct kinematics of the implemented robot.

Consider a static Cartesian reference frame $m=\left(x_{m},y_{m}\right)$ and an auxiliary reference frame $r=\left(x_{r0},y_{r0}\right)$ , given by the motion of the robot. The geometric and locomotion centers of the robot coincide with the origin of *r* (Figure 1). Based on these coordinates, the linear velocity v and the angular velocity $\omega =\left(\omega_{d},\omega_{i}\right)$ can be defined. Where $\omega_{d}$ corresponds to the angular velocity given by the set of actuators on the right side and $\omega_{i}$ to the angular velocity given by the actuators on the left side of the robot. It is important to note that these actuators are synchronized, given the requirements of the differential robot.

The velocity expressions in their matrix form are given by Equation 1.

$${\left[\begin{array}{cc} v & \omega \end{array}\right]}^{T}=A_{r0}{\left[\begin{array}{cc} \omega_{i} & \omega_{d} \end{array}\right]}^{T}$$ (1)

where the velocities v and ω are given by the average of the angular velocities of the actuators due to their effect on the linear as well as angular motion of the robot. The matrix *A*_*r0*_ is shown in Equation 2.

$$A_{r0}=\left[\begin{array}{cc} \frac{r}{2} & \frac{r}{2}\\ \frac{-r}{2L} & \frac{r}{2L} \end{array}\right];\;\left(r>0\right)\wedge \left(L>0\right)$$ (2)

where *r* and 2*L* parameterize the radius of the wheels and the distance between the wheels (left and right), respectively. Note that the matrix $A_{r0}$ is not singular unless the constraint $\left(r>0\right)\wedge \left(L>0\right)$ is not satisfied.

On the other hand, the generalized coordinates of the robot’s position in its plane of motion are defined by Equation 3.

$${\epsilon =\left[\begin{array}{ccc} x & y & \phi \end{array}\right]}^{T}$$ (3)

The relationship between the generalized position coordinates and their derivatives, together with the robot control inputs, is of the form shown in Equation 4.

$$\dot{\epsilon }=A{\left(\epsilon \right)}_{nxm}u$$ (4)

This, in turn, relates to the linear and angular velocity of the robot. The vector of inputs is given by Equation 5.

$$u={\left[\begin{array}{cc} v & \omega \end{array}\right]}^{T}={\left[\begin{array}{cc} u_{1} & u_{2} \end{array}\right]}^{T}$$ (5)

While matrix $A\left(\epsilon \right)$ is shown in Equation 6.

$$A\left(\epsilon \right)=\left[\begin{array}{cc} \cos\left(\phi \right) & 0\\ sen\left(\phi \right) & 0\\ 0 & 1 \end{array}\right]$$ (6)

In Figure 1, it is inferred that the function’s $\cos\left(\phi \right)$ and $sen\left(\phi \right)$ correspond to the *x* and *y* coordinates of the linear velocity vector.

### 3.2 Robot mobility

Definition 1 [Maneuverability]: The degree of maneuverability of the implemented robot is defined by Equation 7.

M=E+D (7)

where E represents the number of degrees of freedom that can be controlled by means of the fixed wheels, and D corresponds to the number of degrees of freedom that can be controlled by means of the directional wheels. Since the differential robot does not have directional wheels, then M=E=2 (Mondal et al., 2020).

Definition 2 [non-holonomic conditions]: A non-holonomic constraint is defined by Equation 8.

$$A\left(\epsilon \right)\;\dot{\epsilon }=0$$ (8)

In this case, the robot presents a non-holonomic constraint, expressed in its matrix form by Equation 9.

$$\left[\begin{array}{cc} sen\left(\phi \right) & -\cos\left(\phi \right) \end{array}\right]\left[\begin{array}{c} \dot{x}\\ \dot{y} \end{array}\right]=0$$ (9)

This constraint, together with Definition 1, suggests that the forward, backward and rotation of the robot are strictly dependent on the geometry of the wheels and, the perpendicularity between these and the horizontal plane. Thus, the robot does not have the ability to follow any trajectory instantaneously (Náprstek and Fischer, 2021).

Remark 1 [Orientation of the robot]: From the relationships presented in Equations 4–6 for the $\dot{x}=v\;\cos\left(\phi \right)$ and $\dot{y}=v\;sen\left(\phi \right)$ , the relationship between the angle ϕ and the angular velocity ω can be abstracted as shown in Equations 10–12. Since ϕ is an angular position, its derivative corresponds to the angular velocity ω .

$$\tan\left(\phi \right)=\frac{\dot{y}}{\dot{x}}$$ (10)

$$\phi =\arctan\left(\frac{\dot{y}}{\dot{x}}\right)$$ (11)

By deriving Equation 11 we obtain Equation 12.

$$\omega =\frac{\dot{x}\ddot{y-}\ddot{y}\dot{x}}{{\dot{x}}^{2}+{\dot{y}}^{2}}$$ (12)

## 4 Exact linearization and robot control

Consider the model expressed by Equations 4–6 and a vector of outputs $z={\left[\begin{array}{cc} x_{r0} & y_{r0} \end{array}\right]}^{T}$ defined by the frame of reference r of the robot. $\dot{z}\left(\phi \right)$ corresponds to the velocity vector in the plane of motion; see Equations 13, 14.

$$\dot{z}\left(\phi \right)={\left[\begin{array}{cc} v_{xr0} & v_{yr0} \end{array}\right]}^{T}$$ (13)

$$\dot{z}\left(\phi \right)=\left[\begin{array}{cc} \cos\left(\phi \right) & 0\\ sen\left(\phi \right) & 0 \end{array}\right]\left[\begin{array}{c} v\\ \dot{\phi } \end{array}\right]=\psi \left(\phi \right)u$$ (14)

**FIGURE 2:**
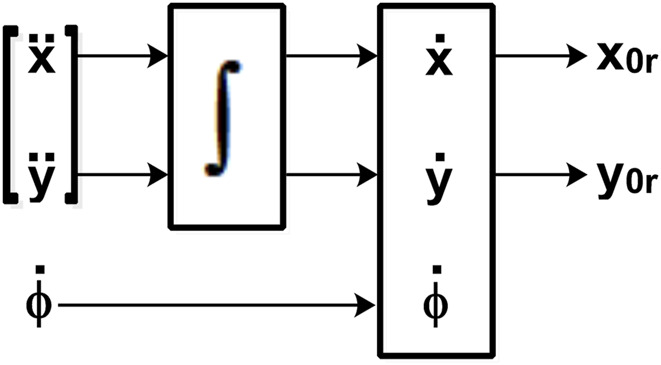
Dynamic extension for system inputs.

Note that the matrix $\psi \left(\phi \right)$ is singular, since it is not invertible. This implies that the system represented by Equation 14 is not controllable and that there is no direct relationship between its inputs and outputs. Therefore, it is necessary to derive a second time according to the realization shown in Figure 2.

Since the system expressed by Equation 14 presents non-controllable states, the scheme shown in Figure 2 is proposed, which incorporates an integrator in the input corresponding to the linear velocity; therefore, for control purposes, this input will be expressed in terms of the linear acceleration ( $\dot{v}$ ) of the system as shown in Equation 15.

$$\ddot{z}\left(\phi ,v\right)=\left[\begin{array}{cc} \cos\left(\phi \right) & -vsen\left(\phi \right)\\ sen\left(\phi \right) & vcos\left(\phi \right) \end{array}\right]\left[\begin{array}{c} \dot{v}\\ \dot{\phi } \end{array}\right]=\varsigma \left(\phi ,v\right)\left[\begin{array}{c} \dot{v}\\ \dot{\phi } \end{array}\right]$$ (15)

where $\ddot{z}={\left[\begin{array}{cc} a_{xr0} & a_{yr0} \end{array}\right]}^{T}$ and the matrix $\varsigma \left(\phi ,v\right)$ is non-singular if and only if v≠0 .

Since $\varsigma \left(\phi ,v\right)$ is invertible, it can be said that there is already a direct relationship between the inputs and outputs of the system; hence, a control input Ʌ can be defined as shown in Equation 16.

$$\left[\begin{array}{c} \dot{v}\\ \dot{\phi } \end{array}\right]=\varsigma^{-1}\left(\phi ,v\right)Ʌ$$ (16)

The above suggests that the relative degree of the controller is equal to two; since Ʌ is the control input, it will impose the desired dynamics. For this reason, the linear relationship between the inputs and outputs of the robot is given by Equations 17, 18.

$$Ʌ=\varsigma \left(\phi ,v\right)\left[\begin{array}{c} \dot{v}\\ \dot{\phi } \end{array}\right]$$ (17)

$$Ʌ=\ddot{z}\left(\phi ,v\right)$$ (18)

Defining the tracking error as the difference between the measured value and the reference, we have Equation 19.

$$e_{z}\left(x_{r0},y_{r0}\right)=z\left(x_{r0},y_{r0}\right)-z^{*}\left(x_{r0},y_{r0}\right)=\left[\begin{array}{c} x_{r0}-x_{r0}^{*}\\ y_{r0}-y_{r0}^{*} \end{array}\right]$$ (19)

Because the relative degree of the controller is equal to two, the control law is a second-order polynomial, as shown in Equation 20.

$$Ʌ={\ddot{z}}^{*}-k_{1}{\dot{e}}_{z}-k_{0}e_{z}$$ (20)

where $k_{1},k_{0}\in R^{+}$ , these correspond to the coefficients of the desired polynomial. On the other hand, the error polynomial is given in Equation 21. If the coefficients $k_{1},k_{0}$ are positive real numbers, then the roots of the desired polynomial are negative and therefore a Hurwitz polynomial (Garzón-Castro et al., 2018).

$${\ddot{e}}_{z}+k_{1}{\dot{e}}_{z}+k_{0}e_{z}=0$$ (21)

Remark 2 [controller constraint handling]: Because the controller is non-singular with ≠0 , in order not to break this constraint, the control algorithm must include a conditional block that avoids division by zero. Another way to address this problem is by handling saturations.

### 4.1 Flat filter approach

The term flat filtering was introduced by Sira-Ramírez (2016), who performed a reinterpretation of Generalized Proportional Integral (GPI) control in terms of classical compensators. The GPI approach seeks to eliminate the dependence on asymptotic state observers from the implementation of integrators (Sira-Ramírez, 2017).

Although the control law shown in Equation 20 is implementable, it strictly requires knowledge of the output and its derivatives, which implies a high implementation cost. Therefore, this section proposes the development of a linear plane filter equivalent to this control law from the perspective of the Generalized Proportional Integral (GPI) control technique. The design sought to avoid the use of state observers.

**FIGURE 3:**
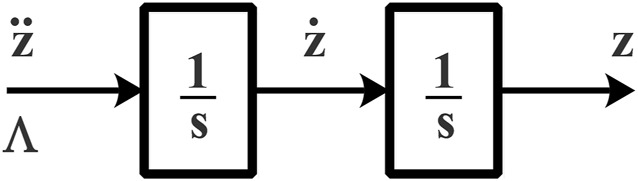
Linearized system dynamics.

The linearization of the robot model required a dynamic extension that decoupled the original linear velocity inputs by linear acceleration inputs. These dynamics of the linearized model shown in Equation 18 can be generalized in the complex frequency domain by the realization shown in Figure 3.

For this case, it was assumed that ${\dot{e}}_{z}$ is a parameter that is not available, since it implies the calculation of the derivative of both the output and the reference signal. On the other hand, the first derivative of the output vector of the system can be obtained by integrating Equation 18.

$$\int_{0}^{t}\frac{d^{2}z\left(\tau \right)}{{d\tau }^{2}}d\tau =\int_{0}^{t}Ʌ\left(\tau \right)d\tau$$ (22)

Solving the integral involving the output of the system, Equation 23 is obtained.

$$\dot{z}\left(t\right)-\dot{z}\left(0\right)=\int_{0}^{t}Ʌ\left(\tau \right)d\tau =\dot{\hat{z}}\left(t\right)$$ (23)

since $\dot{z}\left(0\right)$ is an initial condition, and $\dot{z}\left(t\right)$ is expressed in terms of $Ʌ\left(\tau \right)$ , Equation 23 shows the estimated $\dot{\hat{z}}\left(t\right)$ . Thus, the control law can be expressed as shown in Equation 24.

$$Ʌ={\ddot{z}}^{*}-k_{1}\dot{\hat{z}}\left(t\right)-k_{0}e_{z}\left(t\right)$$ (24)

It would be expected that both acceleration $\ddot{z}$ and velocity $\dot{z}$ would achieve constant steady-state values. Note, however, that when $\dot{z}$ achieves the value of ${\dot{z}}^{*}$ in ≈∞ , the final value ${\ddot{z}}_{f}≅{\ddot{z}}^{*}-k_{0}e_{z}\neq 0$ . Since the steady-state error is nonzero $\dot{\hat{z}}\left(t\right)$ can be said to be an estimate that exhibits estimation errors. This can be corrected by an integral action as shown in Equation 25.

$$Ʌ={\ddot{z}}^{*}-k_{1}\dot{\hat{z}}\left(t\right)-k_{0}e_{z}-k_{i}\int_{0}^{t}e_{z}\left(\tau \right)d\tau$$ (25)

since the objective is to obtain a flat filter, the linear Laplace transform simplifies the integrable terms. Equation 26 groups the Ʌ and $e_{z}$ terms in the complex frequency domain. For this, Equation 22 was replaced in Equation 24.

$$\left(1+\frac{k_{1}}{s}\right)Ʌ={\ddot{z}}^{*}-\left(k_{0}+\frac{k_{i}}{s}\right)e_{z}$$ (26)

Therefore, the control law obtained from the flat filter design is shown in Equation 27.

$$Ʌ={\ddot{z}}^{*}-\left(\frac{k_{0}s+k_{i}}{s+k_{1}}\right)e_{z}$$ (27)

where the coefficients $k_{0}$ , $k_{1}$ and $k_{i}$ must be selected to guarantee the stability of the desired polynomial of the implemented system. Equation 26 is analogous to Equation 20. However, the latter suppresses the derivatives of the implementation, since ${\ddot{z}}^{*}$ can be a pre-feed term.

### 4.2 Tracking trajectories

For the validation of the control law, three desired trajectories were proposed, describing a circumference, a spiral, and finally a trajectory similar to the path that an inspector robot would have to follow through the furrows of a *S. tuberosum* crop. This last trajectory was constructed in sections from the superposition of bounded functions of the hyperbolic tangent type. It is worth noting that for each reference trajectory, it was necessary to determine its first two derivatives since the control law represented by Equation 20 required it. Both the references and their derivatives were established by means of vectors of the form $z^{*}={\left[\begin{array}{cc} f_{x} & f_{y} \end{array}\right]}^{T}$ , where $f_{x,y}$ represents a continuous function for each coordinate axis $\left(x_{m},y_{m}\right)$ . The numerical simulations were performed in Matlab R2023a. Finally, in all cases, the coefficients of the desired polynomial were used $k_{1}=4$ and $k_{0}=8$ . Table 1 shows the mathematical functions that gave rise to each of the trajectories shown with their respective parameters.

**TABLE 1:** Mathematical representation of the implemented trajectories.

Type of trajectory	Mathematical function	Parameters
Circumference	$z^{*}={\left[\begin{array}{cc} \cos\left(\frac{2\pi }{T}t\right) & \sin\left(\frac{2\pi }{T}t\right) \end{array}\right]}^{T}$	T=10 min
Spiral	$z^{*}={\left[\left(o+pt\right)\begin{array}{cc} \cos\left(\frac{2\pi }{T}t\right) & \left(o+pt\right)\sin\left(\frac{2\pi }{T}t\right) \end{array}\right]}^{T}$	o=10 min
		p=10 min
		T=10 min
*S. tuberosum* crop	$z^{*}={\left[\begin{array}{cc} t & 40\sum_{k=0}^{n}{\left(-1\right)}^{k} \end{array}\tanh\left(40t-20k-10\right)\right]}^{T}$	n=20
		T=10 min

Where T represents the period of the function, o corresponds to the initial radius, p to the growth rate of the spiral radius, and n to the number of segments coupled to the function.

**FIGURE 4:**
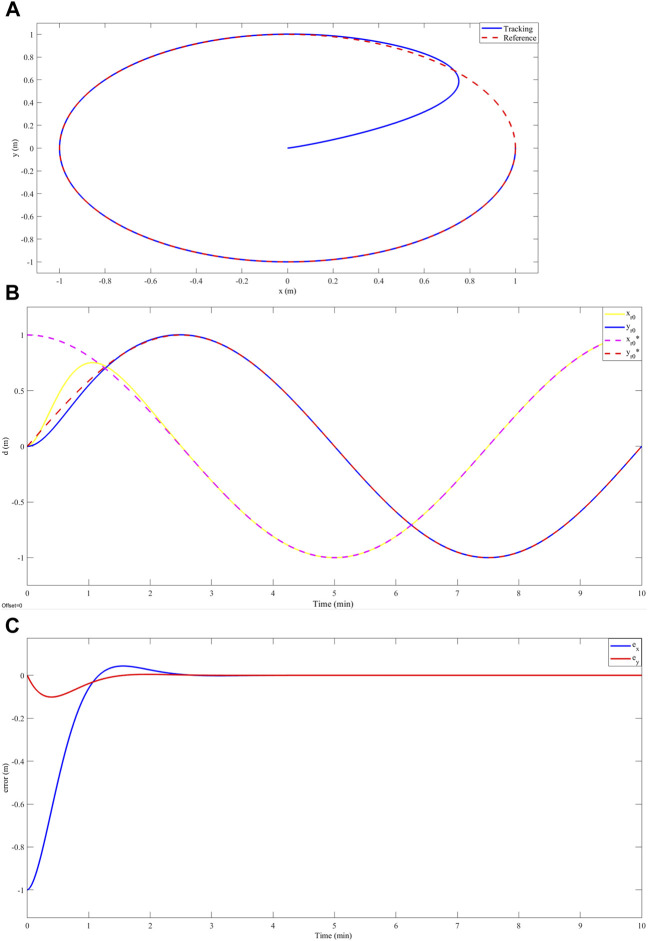
**(A)** Circular trajectory tracking, in red $z^{*}$ and in blue z . **(B)** Tracking the *x* and *y* coordinates of the circular trajectory as a function of time, in yellow $x_{r0}$ , in blue $y_{r0}$ , in purple ${x_{r0}}^{*}$ , and in red ${y_{r0}}^{*}$ . **(C)** Error in the *x* (blue) and *y* (red) coordinates of the circular trajectory as a function of time.

**FIGURE 5:**
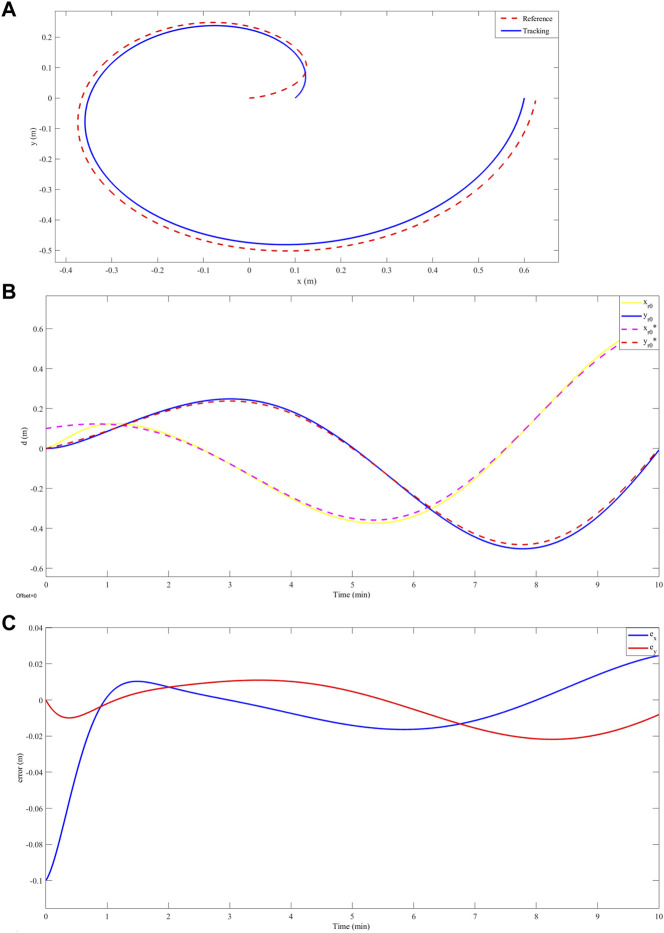
**(A)** Spiral trajectory tracking, in red $z^{*}$ and in blue z . **(B)** Tracking the *x* and *y* coordinates of the spiral trajectory as a function of time, in yellow $x_{r0}$ , in blue $y_{r0}$ , in purple ${x_{r0}}^{*}$ , and in red ${y_{r0}}^{*}$ . **(C)** Error in the *x* (blue) and *y* (red) coordinates of the spiral trajectory as a function of time.

**FIGURE 6:**
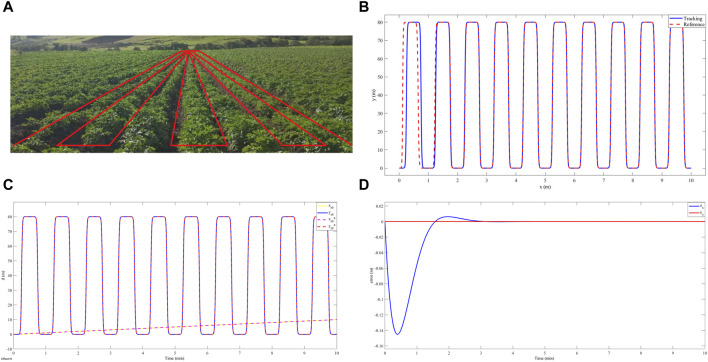
**(A)** Geometry of the robot path in a crop of *Solanum tuberosum* in the department of Cundinamarca, Colombia. **(B)** Tracking of a trajectory like that of a culture of *Solanum tuberosum*, in red $z^{*}$ and in blue z . **(C)** Tracking the *x* and *y* coordinates of the trajectory similar to that of a culture of *Solanum tuberosum* as a function of time, in yellow $x_{r0}$ , in blue $y_{r0}$ , in purple ${x_{r0}}^{*}$ , and in red ${y_{r0}}^{*}$ . **(D)** Error in the *x* (blue) and *y* (red) coordinates of the trajectory like that of a culture of *Solanum tuberosum* as a function of time.

Figure 4 shows the circular trajectory tracking results. A. shows that the controller achieves a correct trajectory tracking in 2.3 min after a maximum overshoot of less than 1% of its nominal value. B. shows that both the overshoot and settling time are given specifically for the x-coordinate of the robot’s trajectory. However, in Figure 4C. A position error equal to zero is evidenced after the settling time. The above shows an asymptotically stable behavior. On the other hand, for a spiral-type trajectory, see Figure 5A illustrates a deviation of less than 3% between the desired trajectory and the trajectory achieved by the robot. Figure 5B shows that this deviation corresponds to both the x-coordinate and the y-coordinate of the motion. Figure 5C shows that there is a position error for the two coordinates of the motion. This position error has an oscillatory characteristic at values lower than 3%. Finally, in relation to the trajectory similar to the path that the robot would follow in a crop of S. tuberosum whose characteristics are illustrated in Figure 6A. Correct tracking was evidenced after an establishment time of 0.9 min (Figure 6B.), at this time, a deviation of less than 0.1 m was evidenced between the reference path and the path achieved by the robot (Figure 6C). Figure 6D shows an error equal to zero in the stable region, which indicates an asymptotically stable behavior.

## 5 Discussion

The feedback linearization technique has been widely used in recent decades for the control of nonlinear systems (Akbarimajd et al., 2019). It cancels the nonlinear terms present in the system and imposes the desired dynamics by means of the coefficients $k_{1}$ and $k_{0}$ (Al-Durra and Errouissi, 2019). These coefficients can be arbitrarily selected if they lead to the construction of a desired polynomial with negative roots. (Astrom and Murray, 2021). The results of this work agree with the literature regarding the advantages that feedback linearization can offer over other algebraic techniques used in RMA, these include: I) versatility, since they can be applied to a wide range of Single Input Single Output (SISO) or Multiple Input Multiple Output (MIMO) nonlinear kinematic models (as is the case of differential robots), II) good performance, since one of the main characteristics is its fast response and stability, a necessary requirement when operating a robot for agricultural inspection, III) applicability, since, once the system is linearized, the controller is an algebraic polynomial whose relative degree depends on the number of derivatives that achieve an input-output relationship, and IV) adaptability, since the exact linearization technique can be articulated with other advanced control techniques such as Generalized Proportional - Integral (GPI) control (Moreno et al., 2023). However, the controllers obtained from the exact linearization generate a high dependence on the robot model, the desired trajectory, and its derivatives. This suggests a high cost in instrumentation and information processing, in addition to the construction of trajectories by bounded sections such as the trajectory that a robot inspector of S. tuberosum crops would perform (not reported in other works). On the other hand, the control technique using exact linearization often leaves the controllers vulnerable to the effects of external perturbations from the environment and changes in the robot parameterization, as suggested by Al-Durra and Errouissi (2019). In recent years, alternative approaches have been proposed that can result in controllers equivalent to those obtained classically. However, the advantages of these equivalent designs lie in their lack of dependence on output derivatives and state observers.

Regarding the feedback linearization method, this work shows a simplified version that agrees with the results reported by Sarkar et al., (1994) y Kim and Oh (1999), which have served as a reference in the AMR paradigm. However, we present a linear filter equivalent to the classical design reported in the literature. This filter shown in Equation 27 suggests more practicality in the implementation since it corresponds to a transfer function that discards the derivatives of the robot output, unlike the classical design shown in Equation 20.

Our results showed accurate tracking on a circular trajectory with a settling time of 2.3 min and an overshoot of less than 1% (Figure 4). While, in the work developed by Sira-Ramírez (2017) reported the design of a robust controller from the Active Disturbance Rejection Control (ADRC) approach for an omnidirectional robot, this work showed accurate tracking without overshoot for a circular trajectory in a time of 2.5 min. This can be attributed to the fact that this type of robot can track the reference with less control effort since it has an additional degree of freedom compared to the differential robot. Furthermore, Tilahun et al. (2023) presented a fuzzy adaptive sliding mode control for a differential robot and made a comparison of this strategy with classical PID control for tracking a circular trajectory. Their results showed amplitude oscillations of less than 0.5% around the reference trajectory. This is evidence that our design, having an asymptotic response, presents better trajectory tracking. On the other hand, to evaluate our controller against trajectories with abrupt variations in the x and y coordinates, we report the tracking of the differential robot to a spiral-type trajectory. In this case, our robot did not achieve the reference values and presented a constant deviation of less than 3% without overshoot (Figure 5). Luviano-Juárez et al. (2015) addressed the problem of tracking a two-wheel differential robot to a flower-shaped trajectory by designing a controller obtained with the pre-feed linearization strategy and differential flatness, showing accurate tracking in their numerical results. However, its implementation presented a constant tracking error of less than 3%. Among other works, Mondal et al., (2020) performed a detailed analysis of the maneuverability of a differential robot and proposed a tracking control for a trajectory with abrupt curves using predictive control strategies, for which deviations of less than 2% were observed between the trajectory achieved by the robot and the changes given by each curve. These variations can be attributed to the movement restrictions of the robot, since it lacks the capacity for lateral movements. Finally, for trajectories associated with the geometry of an S. tuberosum crop, we report a trajectory constructed by segments of hyperbolic tangent functions, for which correct tracking is observed after a setup time of 3.2 min and a maximum overshoot of 5% (Figure 6). Regarding the same trajectory, Heikkinen et al., (2017) presented a fuzzy PID controller implemented on a differential robot for tracking a straight line, showing tracking improvements over classical PID control. However, this fuzzy PID controller shows a 10% deviation between the achieved trajectory and the reference. These results show that our controller presents improvements in terms of accurate tracking of the reference.

In the face of the basic requirements for tracking terrestrial trajectories associated with agricultural crops, such as S. tuberosum, the controller obtained in this work can provide good performance if there are no abrupt changes in reference coordinates, as in the case of linear or circular trajectories. The controller obtained in this work can provide good performance if there are no abrupt changes in the reference coordinates, as in the case of linear or circular trajectories. When following spiral trajectories, it is suggested that the *o* and *p* parameters directly influence the motion constraints given in Section 2. Since the wheel arrangement is fixed and the wheels in turn lack directionality, the robot cannot make abrupt lateral movements or follow instantaneous changes in the *x* and *y* coordinates, as could occur if the wheels were spherical (Náprstek and Fischer, 2020).

## 6 Conclusion

A controller via exact linearization was designed for a differential robot inspector of *S. tuberosum* crops in the department of Cundinamarca, Colombia. The objective of this controller was to track desired ground trajectories. The analysis of the robot’s motion constraints added to its non-linear behavior resulted in a system with two degrees of mobility and a non-holonomic constraint. These constraints allowed accurate tracking of trajectories with constant trends in both x and y coordinates. Likewise, the robot lacks the ability to track trajectories with abrupt changes in any of the coordinates. The implementation of the linear control law shown in this work suggests a reliable option that satisfies some basic requirements for tracking agricultural land trajectories. However, although the controller solves some stability and tracking problems, it is highly sensitive to perturbations in the scanning environment and parametric changes in the robot. For this reason, future work is expected to apply disturbance rejection techniques to obtain a controller that is more robust to such variations.

## Data Availability

The raw data supporting the conclusions of this article will be made available by the authors, without undue reservation.
